# A robust class decomposition-based approach for detecting Alzheimer’s progression

**DOI:** 10.1177/15353702231211880

**Published:** 2023-12-07

**Authors:** Maha M Alwuthaynani, Zahraa S Abdallah, Raul Santos-Rodriguez

**Affiliations:** 1 University of Bristol, Bristol BS8 1TH, UK; 2 College of Computer Science & Information Systems, Najran University, Najran 61441, Saudi Arabia

**Keywords:** Structural MRI, class decomposition, transfer learning, Alzheimer’s disease, mild cognitive impairment

## Abstract

Computer-aided diagnosis of Alzheimer’s disease (AD) is a rapidly growing field with the possibility to be utilized in practice. Deep learning has received much attention in detecting AD from structural magnetic resonance imaging (sMRI). However, training a convolutional neural network from scratch is problematic because it requires a lot of annotated data and additional computational time. Transfer learning can offer a promising and practical solution by transferring information learned from other image recognition tasks to medical image classification. Another issue is the dataset distribution’s irregularities. A common classification issue in datasets is a class imbalance, where the distribution of samples among the classes is biased. For example, a dataset may contain more instances of some classes than others. Class imbalance is challenging because most machine learning algorithms assume that each class should have an equal number of samples. Models consequently perform poorly in prediction. Class decomposition can address this problem by making learning a dataset’s class boundaries easier. Motivated by these approaches, we propose a class decomposition transfer learning (CDTL) approach that employs VGG19, AlexNet, and an entropy-based technique to detect AD from sMRI. This study aims to assess the robustness of the CDTL approach in detecting the cognitive decline of AD using data from various ADNI cohorts to determine whether comparable classification accuracy for the two or more cohorts would be obtained. Furthermore, the proposed model achieved state-of-the-art performance in predicting mild cognitive impairment (MCI)-to-AD conversion with an accuracy of 91.45%.

## Impact Statement

It is challenging to predict whether individuals with mild cognitive impairment (MCI) will develop Alzheimer’s disease (AD). Therefore, our investigation concentrated on predicting which patients will progress from MCI to AD. Using the class decomposition transfer learning (CDTL) model to detect Alzheimer’s progression opens up new possibilities for research in the field of developing and designing tools to assist clinicians in identifying individuals at risk for AD and those with the earliest signs of clinical impairment, which is critical for treating and preventing AD.

## Introduction

About 60–80% of dementia cases are caused by Alzheimer’s disease (AD) whose patients experience a range of symptoms, which change over time and correspond to the degree of neuronal damage in different brain regions.^1^ AD is characterized by decreased memory, loss of thinking and reasoning skills, and changes in behavior that interfere with daily life. These difficulties result from damaged or destroyed nerve cells in brain regions involved in cognitive function and essential physical functions, such as walking. Years pass before this disease manifests symptoms, and each person’s rate of symptom progression from mild to moderate to severe varies.^1^ Early detection of AD progression can offer enormous opportunities for proactive interventions to delay the disease’s progression. Mild cognitive impairment (MCI) is a clinical condition that occurs between normal aging and AD, in which people experience more memory loss than one would expect for their age.^2^ People with MCI have mild changes in cognitive ability, which will be apparent to affected individuals and others; however, they can continue to engage in their everyday activities. Approximately 15–20% of individuals aged 65 years or older have MCI, and 30–40% of these will develop AD within 5 years; thus, not all patients with MCI will develop AD.^1^ MCI patients develop clinically probable AD much faster than healthy people their age. Most MCI patients could progress to AD at a rate of 10–15% annually, while healthy control (HC) subjects could convert at a rate of 1–2% per year.^2^ Therefore, MCI should be recognized and further researched because clinicians will be needed to identify individuals at risk for AD and those with the earliest signs of clinical impairment.^2^ MCI patients can be classified as MCI converters (MCIc) or MCI non-convertors (MCInc), indicating that they do or do not convert to AD within the typical conversion period, ranging from 6 to 36 months, with an average of 18 months.^1^

It has been proven that neuroimaging techniques are significantly helpful in the diagnosis of AD. The most widely used neuroimaging technique for identifying AD is structural magnetic resonance imaging (sMRI), which can identify both the white matter and gray matter atrophy caused by the loss of white matter tract integrity and neurons and synapses in Alzheimer’s patients. Considerable progress has recently been made in the detection and monitoring of AD progression using brain sMRI data.^3^

Deep learning has attracted significant interest in Alzheimer’s detection research since 2013, with the number of publications on the subject increasing dramatically since 2017.^3^ Many studies have assessed structural brain variances to highlight the atrophy of AD and prodromal AD, many of them are using a voxel-based method which is based on Voxel intensity values from the entire neuroimaging modality. Approximately 70% of studies employing this approach include a full-brain analysis.^3^ The acquisition of three-dimensional (3D) data from neuroimaging scans is achievable by fully integrating the spatial data, which is the main benefit of whole-brain analysis. The disadvantage of using a voxel-based approach is that it increases the dimensionality of data and the computational load.^4^ The voxel-based approach has been used in numerous studies with a variety of techniques.^5,6^ A deep learning model with two-dimensional (2D) data typically has fewer parameters and requires less training time than when using 3D data. To reduce the number of hyperparameters, some studies used 2D slice-based approaches to obtain 2D slices from 3D brain scans. Using a slice-based approach could lead to a loss of information by reducing volumetric data to 2D representations because actual brain tissue is represented in 3D images.^3,4^ As a consequence of using the slice-based technique, a whole-brain analysis is not achievable.^3^ Many studies have used unique methods to extract 2D image slices from 3D brain scans, while others have used standard projections of the axial, sagittal, and coronal planes.^7,8^

Several studies have used neuroimaging and efficient machine learning techniques to differentiate between AD, MCI patients, and cognitively normal (CN).^7,9–11^ However, it is still challenging to distinguish between MCI patients who progress to AD (pMCI) and those who remain stable without progressing to AD (sMCI).^2^ According to some studies,^12,13^ one approach for addressing the classification of sMCI and pMCI classes is to train the model on mixed cohorts of CN, AD, pMCI, and sMCI subjects. Furthermore, the pMCI and sMCI classes cannot be considered independent. Therefore, some studies suggested a strategy used to address the classification of sMCI and pMCI classes, which is to employ a pretrained network trained on diverse cohorts, including CN, and AD to predict whether or not people with MCI will convert to AD.^14–16^

Basaia *et al.*^14^ used a 3D convolutional neural network (3D CNN) based on a single cross-sectional sMRI to predicate MCI to AD conversation. In addition, this study investigated the limitations of using a single-center dataset. The researchers used two datasets, ADNI (ADNI 1/2/GO) and Milan, for training, validating, and testing the 3D CNN for six binary classifications. The model’s performance was evaluated first on the ADNI dataset and then on the combined dataset (ADNI and Milan). Transfer learning was employed to enhance the performance of classifiers. To decrease training time and improve efficiency, the weights from the CNN used to classify ADNI AD and HC subjects (AD versus HC) were used as a pretrained network in other CNNs to classify the remaining classes. As a result, CNNs achieved an accuracy of 75% for differentiating patients with MCI converters (cMCI) from those that will remain stable (sMCI), with no differences between ADNI and non-ADNI image accuracy. In Pan *et al.*,^15^ the authors suggested the (CNN-EL) approach, which combines CNN and Ensemble Learning to be used in three binary classification tasks to distinguish between subjects with MCIc and MCInc and those with AD and MCI from HCs. The researchers used a set of sagittal, coronal, or axial MRI slices taken from the subjects in the dataset at a specific brain location to create a 2D CNN model. First, each set of sagittal, coronal, or axial MRI slices was used in training base classifiers. Then classifier ensemble based on single-axis slices was created, and the trained base classifiers with optimal performance on the validation datasets were chosen and combined to generate an enhanced final classifier ensemble based on three-axis slices. The CNN-EL model achieved a 62.0% accuracy in distinguishing patients with MCI converters from those who will remain stable. Bae *et al.*^16^ used a CNN model that was first trained on sMRI scans of healthy individuals and those with AD to predict which individuals with MCI converted (MCI-C) and which did not convert (MCI-NC). Researchers used a classification task of NC versus AD as the source task for transfer learning to the target task, which is MCI-C versus MCI-NC. The knowledge gained from the source task is transferred to the target task, which employs scans from MCI patients. The model is then retrained using scans from MCI-NC and MCI-C patients to extract features that can predict AD conversion. The model’s accuracy in predicting MCI-to-AD conversion was 82.4%. For various reasons, research studies^14,16^ classify sMCI and pMCI using a model trained to differentiate between AD patients and HCs. It is more difficult to distinguish between sMCI and pMCI than between AD and CN. The classification of sMCI versus pMCI is similar to CN versus AD. The knowledge gained from training a model to recognize HCs from AD patients can be helpful when fine-tuning that model to distinguish between the sMCI and pMCI.

Training the model from scratch has some limitations. The main limitation is that training models require an extensive amount of labeled data. Transfer learning can be employed as an alternative to training the model from scratch.^17^ A crucial machine learning technique for addressing the issue of insufficient training data is transfer learning which transfers knowledge from one domain to another.^18^ Another limitation of using deep learning on sMRI data is that model training requires significant computational resources. Furthermore, dealing with irregularities in dataset distribution is challenging. Class decomposition contributes to solving the issue of irregularities in dataset distribution by assisting in learning the class boundaries of a dataset. The class decomposition method breaks down each class into subclasses, which are treated independently to deal with any irregularities in the data distribution.^10^

In this article, we assess the robustness of the class decomposition transfer learning (CDTL) model. A model might achieve promising performance; however, this performance may be fallacious, that is, a model may learn from disease-irrelevant confounders in the training data to attain high performance on test sets. As a result, to be confident in the model’s results, it is necessary to assess its robustness. In this article, we examine the model’s robustness using data from various cohorts of sMCI and pMCI subjects. Our study’s objectives are to: (1) determine whether comparable classification accuracy for the two or more cohorts would be obtained and (2) evaluate the prediction accuracy of using the combined cohorts.

## Materials and methods

### Participants and datasets

Data used in the preparation of this article were obtained from the Alzheimer’s Disease Neuroimaging Initiative (ADNI) database (adni.loni.usc.edu). The ADNI was launched in 2003 as a public-private partnership, led by Principal Investigator Michael W. Weiner, MD. The primary goal of ADNI has been to test whether serial magnetic resonance imaging (MRI), positron emission tomography (PET), other biological markers, and clinical and neuropsychological assessment can be combined to measure the progression of MCI and early AD.

We use the sMRI from the ADNI1, ADNI2, and ADNI-GO phases. ADNI-1 initial phase began in October 2004. The ADNI1 study collected and analyzed tens of thousands of brain scans, genetic profiles, blood, and cerebrospinal fluid biomarkers with the original goal of discovering more sensitive and exact biomarkers for the early detection and tracking of AD. A total of 200 elderly control subjects, 200 participants with early AD, and 400 participants with MCI were included in the study. In 2009, ADNI-1 was expanded during the Grand Opportunities (ADNI-GO) phase, which evaluated 200 additional participants with early mild cognitive impairment (EMCI) in addition to the existing ADNI1 cohort. Examining biomarkers at an earlier stage of the disease was the goal of this phase. In addition, MR protocols were modified during the ADNI-GO phase. ADNI2 started in 2011 and evaluated participants from the ADNI1/ADNI-GO phases as well as the new participant groups listed: 150 elderly controls, 100 people with EMCI, 150 people with late mild cognitive impairment (LMCI), and 150 people with mild AD. To close the gap between HCs and MCI, the significant memory concern (SMC) cohort was also added to ADNI2. A participant’s self-reported SMC is a crucial inclusion criterion. A total of 107 SMC subjects were included in the study.^19^

We utilize the sMRI from the ADNI1, ADNI2, and ADNI-GO phases to construct two different datasets. The first dataset for building the base classifier, which was used to train on AD and HC subjects. This model will be used later to retrain MCI patients to recognize between sMCI and cMCI. We follow the same strategy followed by some studies^14–16^ in the literature to address the classification of sMCI and pMCI classes: to employ a pretrained network trained on AD versus healthy individuals to predict whether or not people with MCI will convert to AD. The first dataset contains 89 3D T1-weighted magnetic resonance images from 89 subjects (45 patients with AD and 44 HCs), whereas the second dataset includes (467) 3D T1-weighted baseline sMRI scans for patients with probable MCI to track the progression of MCI conversion in more detail. Based on the follow-up period (36 months) for all subjects, MCI subjects were divided into 146 pMCI and 321 sMCI. The subjects’ diagnosis remained the same throughout the entire period of follow-up. The demographic characteristics of the subjects at baseline are illustrated in Tables 1 and 2.

**Table 1.** Characteristics of cognitively normal (CN) and Alzheimer’s disease (AD) participants.

Characteristic	CN	AD
Subjects	44	45
Gender (M/F)	22/22	21/24
Age range (mean, SD)	61–89 (75.18 ± 7.10)	62–90 (74.75 ± 7.94)
MMSE (Mini Mental State Examination) (mean, SD)	27–30 (29.19 ± 0.94)	20–26 (23.15 ± 2.19)

SD: standard deviation.

**Table 2.** Characteristics of mild cognitive impairment (MCI) participants.

Characteristic	ADNI1	ADNI2	ADNIGO
Subjects (sMCI/pMCI)	94/83	150/56	77/7
Gender (M/F)	58/77	86/120	46/38
Age range (mean, SD)	55–87 (74.37 ± 7.38)	55–88 (70.65 ± 7.13)	55–88 (71.25 ± 7.30)
MMSE (Mini Mental State Examination) (mean, SD)	23–30 (27.19 ± 1.70)	24–30 (27.95 ± 1.70)	23–30 (28.21 ± 1.64)

ADNI: Alzheimer’s Disease Neuroimaging Initiative; ADNIGO: Alzheimer’s Disease Neuroimaging Initiative Grand Opportunities; SD: standard deviation; sMCI: stable MCI; pMCI: progressive MCI.

Our selection criteria concentrated on subjects that underwent follow-up exams 3 years after their baseline. AD and CN had the same diagnosis for the entire 36-month follow-up period, every subject whose diagnosis changed from the baseline was excluded. For all MCI subjects diagnosed with MCI in the baseline and during the 36-month follow-up period, any subject reversions to CN during the 3 years were excluded. The subjects with sMCI had been diagnosed with MCI and had not had any conversions to AD or reversions to CN during the 3 years (36 months) following their baseline visit, whereas the subjects with pMCI converted to AD within 3 years.

The sMRI (T1-weighted MPRAGE at 1.5 T in ADNI1 and 3 T in ADNI 2/GO) was downloaded in the Digital Imaging and Communications in Medicine (DICOM) format and converted to the Neuroimaging Informatics Technology Initiative (NIfTI) format using the MRIcron software (https://www.nitrc.org/projects/mricron). Before being downloaded, scans were corrected for bias-field inhomogeneities as part of the ADNI preprocessing protocol. Then, images were processed and analyzed using FSL tools and Python libraries, and preprocessing included skull stripping, smoothing, and co-registration to Montreal Neurological Institute (MNI) space, which resulted in images with 197 × 233 × 189 voxels and a spatial resolution of 1 × 1 × 1 mm^3^. We align each subject’s brain to the MNI template to ensure that specific brain regions, such as the hippocampus, are consistently included in 2D slices across all subjects. This alignment allows extracting various subjects’ slices with the same regions. Following the procedures, axial image slices of each subject’s 3D image were created and saved in the Joint Photographic Experts Group (JPEG) format.

### Selection of the most informative 2D dataset

It can be challenging to handle high-resolution 3D images, particularly in terms of data volume, computational complexity, and storage. The volume of 3D MRI scans is one of the major challenges. Each 3D MRI scan consists of several 2D slices that have been stacked together to capture details from various perspectives and depths to form a 3D representation, resulting in a significantly larger image size when compared with a single 2D image. Furthermore, processing 3D data requires more computational resources than processing 2D data, resulting in longer processing times that slow down training and increase the cost of the process. Deep learning model process 3D data demands more computational resources than their 2D counterparts. Furthermore, the voluminous nature of 3D MRI data requires massive storage requirements. Using 2D slices rather than 3D images can address these challenges.3 When training deep learning models, especially with limited computational resources, it is essential to use slices that contain the most relevant information.

The 3D sMRI of each subject is re-sliced into a 2D scan. Extracting 2D slices produces a vast number of images, not all slices in a 3D volume are equally informative.^3^ Some of them contain noisy data, while others are rich in information. Therefore, the most informative slices are extracted and used to train and test the model in our suggested method.

Typically, the center of the 3D brain scan contains more data than the edges. The images in the center have a higher information entropy than the other regions in the images. Entropy can provide information about the texture and distribution of pixel values in an MRI image. High entropy values indicate that the image contains a range of pixel intensities, implying more detail or complexity. Lower entropy values indicate more uniform regions, indicating a different tissue type or a lack of pathology.^3^ In consideration of this, we choose slices based on the image entropy which can be calculated using the gray-level co-occurrence matrix (GLCM)^20^ for a single slice. GLCM is a second-order statistical method for texture analysis. It examines the spatial relationship among pixels of an image. By examining how often pairs of pixel values co-occur at a given offset, GLCM captures intricate patterns and structures in an image. Using a co-occurrence matrix, this method extracts textural information about the gray-level transition between two adjacent pixels.^20^ The spatial relationship between two adjacent pixels, defined by direction θ and distance *D*, can be used to calculate various matrices such as contrast, homogeneity, energy, and entropy that provide various information.^20^

We apply two different methods to select the most informative 2D slices. On the first dataset used to train and test the base classifier, we use the image entropy method. Following the extraction of all 2D axial slices for each subject, we use the GLCM to determine the image entropy for each slice. Some studies select the most informative slices using an entropy-based sorting procedure, which relies on sorting the slices after calculating image entropy because the most informative slices have the highest entropy values.^3^ Following the current studies, the 2D images were sorted in descending order based on the entropy, images with the greatest entropy values are the most informative. The 2D slices in the center of the 3D image have the highest image entropy in comparison with the other parts of the image, as shown in Figure 2. For the model’s training and testing, we chose 20 slices with the highest entropy from each subject. In addition, based on the assumption made by some recent studies that the middle slices cover the areas that have the essential features for the classification task,^3^ which are the hippocampus, cortex, and ventricles, we used the technique of selecting the middle slices of the 3D images in order to form the 2D dataset for the MCI classification task. This technique reduces the computational complexity required to process the volume of volumetric data while providing valuable data for deep learning models. We extracted the 30 middle slices of each 3D image for the second dataset. The middle slices for the MCI dataset were determined based on the total number of slices in each 3D image, ensuring a central starting point. Given that all our subjects underwent the same scanning protocol, the middle slice is consistent in its anatomical representation across subjects.

### Feature extraction and classification

**Figure 1.**
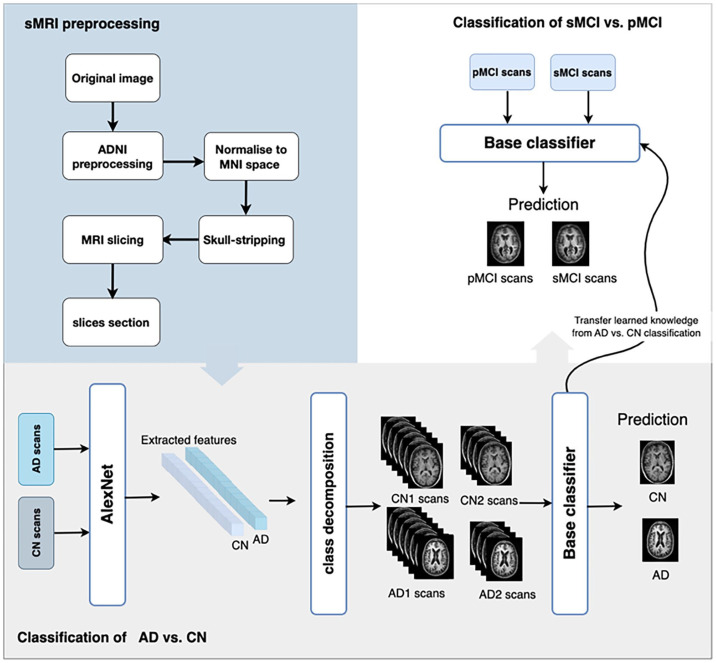
The overall architecture of the CDTL (class decomposition transfer learning) model. THE ADNI structural MRI images are re-sliced after preprocessing and alignment to MNI space to select the most informative slices. Next, extract the features from the scans of cognitively normal (CN) individuals and Alzheimer’s disease (AD) patients using AlexNet, then pass these features to the class decomposition algorithm and VGG19 to train and create the base classifier. Then, the base classifier is used as a pre-trained model to distinguish between sMCI and pMCI by freezing all of its layers and modifying the top layer for the new classification task.

The model used to build the base classifier, which is used to train on AD and HC subjects is inspired by Abbas *et al.*^21^ Images for all subjects go through three phases: feature extraction, class decomposition, and classification. Transfer learning is used in both the feature extraction and classification phases. Figure 1 illustrates the proposed model using sMRI.

**Figure 2.**
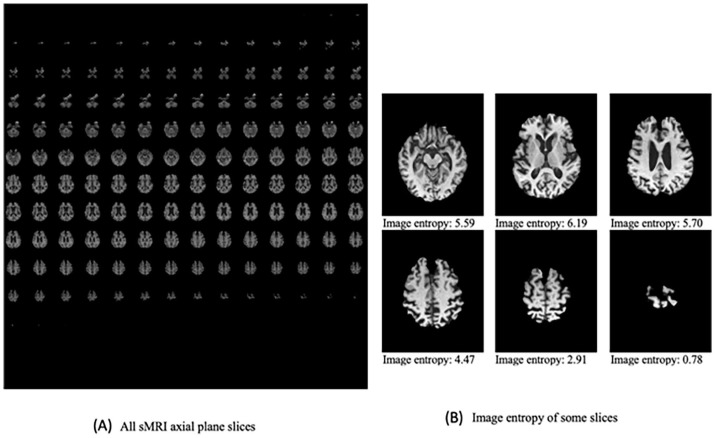
(A) Extracting all two-dimensional axial slices of a 3D sMRI shows that this process generates many 2D images, some containing noisy data while others are rich in information. (B) The two-dimensional axial slices in the centre of the sMRI scan have the highest image entropy compared to the other regions, indicating that the most useful information is contained in the images with the highest entropy.

For feature extraction, we use a pretrained model on ImageNet to capture the general features from images by freezing some layers of the pretrained network and adapting the top layer to be used for ADNI data. AlexNet network is used to extract features from 2D images. The top layer of the pretrained network is adopted for the two classes AD and CN. After extracting the features, we used principal component analysis (PCA) to reduce the dimensionality of the feature space, which assists in reducing memory requirements and enhancing the framework’s efficiency. Then, the extracted features were passed to the cluster to perform the class decomposition. Applying clustering as a preprocessing phase for each class is known as class decomposition which was proposed by Vilalta *et al.*^22^ The idea of the clustering-based class decomposition approach is that clustering is applied to all data samples of each class to divide the class into clusters (subclasses) and to re-label each cluster’s instances with a new class label. This technique assists in decreasing the impact of noisy data, discovering the hidden patterns within each class, and enhancing classification accuracy.^22^ Following the class decomposition, VGG19 is used for the classification task in the classification phase. The VGG19 network was trained on the new dataset that resulted from class decomposition. Depending on the dataset before decomposition, the predicted class of VGG19 (subclasses) will be reassembled to construct the original classes before decomposition.

After training the base classifier on predicting AD versus CN, it is used as a pretrained model to differentiate between sMCI and pMCI by freezing all its layers and adapting the top layers for the new classification task.

2D scans from 89 subjects (45 AD and 44 CN) are used to build the base classifier. In order to classify AD versus CN and transfer the learned weights to predict who will develop AD. We experimented with AlexNet to extract the features from 2D images. First, by fine-tuning the network to extract the features, the top 13 layers of the AlexNet network are adopted for the two classes. A tuned network is then used to extract the features. The AlexNet was trained using 200 epochs, 50-batch sizes, and Adam as an optimizer with learning rates of 0.0001. We then scale the features after using PCA to reduce the dimensionality. In the class decomposition, the features of each class, CN and AD, are divided into two subclasses using a K-means cluster with *k* = 2. Then we use VGG19, a pretrained network on ImageNet, to classify AD versus CN. The top two layers of the VGG19 are adjusted for the four classes produced by the class decomposition step. Ten-fold cross-validation is used to train and validate the model.

The pretrained model and its weight are transferred to classify sMCI versus pMCI. The pretrained network is used by freezing all its layers and replacing the top layer to be used as a binary classifier in the new classification task. For each subject in the four different datasets – ADNI-1, ADNI-2, ADNI-GO, and combined cohort datasets (ADNI-1, ADNI-2, and ADNI GO) – the network is retrained using 10-fold cross-validation and 30 2D scans from the center of the 3D image.

## Results

This section presents the findings from the base classifier and the pretrained model used to differentiate between sMCI and pMCI. A total of 556 subjects (45 AD, 44 CN, and 467 MCI) were used in the datasets, which were randomly split into 90% training sets and 10% test sets based on the subjects. Accuracy, precision, recall, and the F1-score were used to assess the performance of both models.

### Base model’s results

The base model is employed as a pretrained network to distinguish pMCI from sMCI within 36 months after being used to classify Alzheimer’s patients and healthy individuals. We employed the silhouette score to assess the effectiveness of the clustering phase of the base model. The average silhouette score for the CN class is 0.99, while the AD class receives a score of 1. Also, several evaluation metrics demonstrate the model’s remarkable performance, as shown in Table 3. The model’s accuracy, which was 98.5%, indicated that it typically predicted the cohort’s participation correctly. An in-depth analysis of the performance metrics revealed that the model correctly identified 99.0% of the true AD cases with a recall of 99.0%. These metrics show that the model is highly accurate and reliable in differentiating between the AD and CN cohorts.

**Table 3.** Performance metrics of the base model using healthy individuals and Alzheimer’s patient datasets.

Metric	Value
Accuracy (%)	98.5%
Sensitivity (%)	99.0%
Specificity (%)	97.0%

### Pretrained model’s results

**Figure 3.**
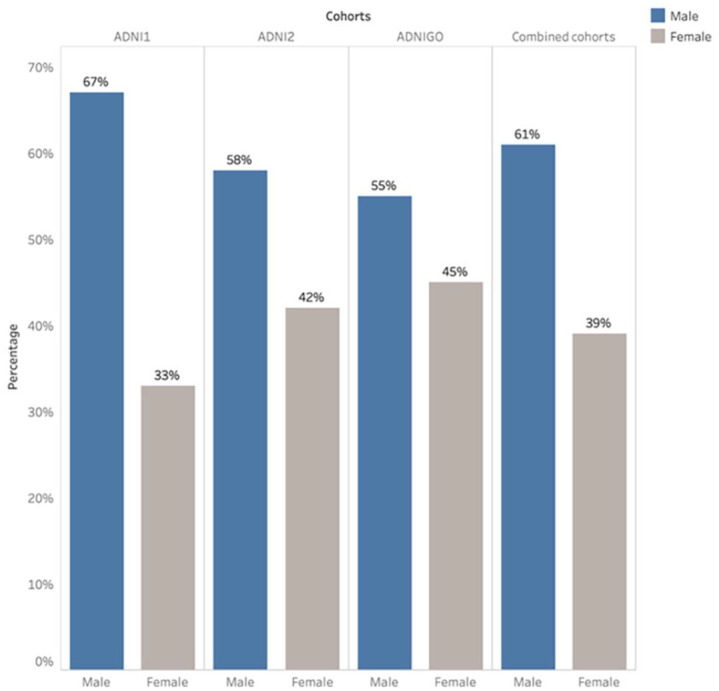
The ratio of male to female within each MCI cohort.

**Figure 4.**
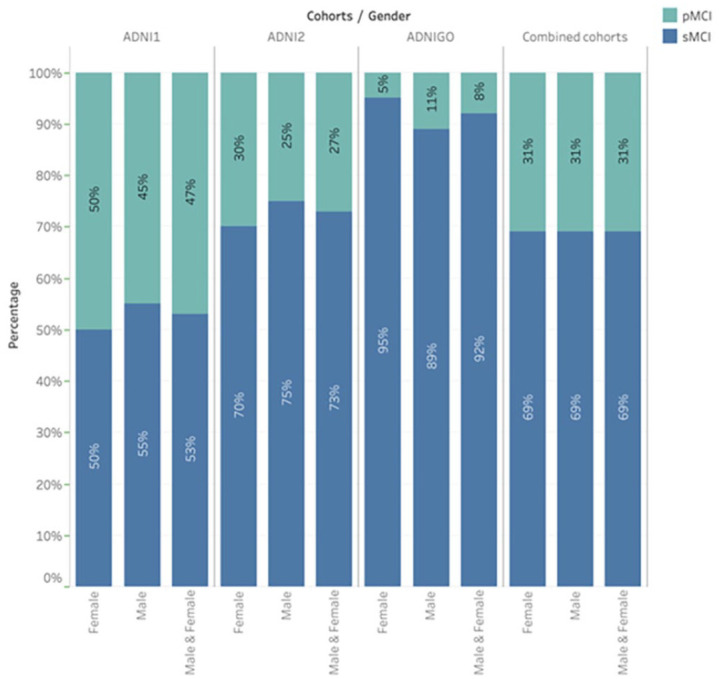
The ratio of sMCI to pMCI within each MCI cohort.

A pretrained model is used to differentiate between sMCI and pMCI. Four different datasets – ADNI1, ADNI2, ADNI-GO, and a combination of all these cohorts (“combined cohorts”) – are used in the experiments. Within each MCI cohort, the ratios of male to female and sMCI to pMCI are shown in Figures 3 and 4. The ADNI1 cohort includes 67% male and 33% female subjects. Men experience sMCI in 55% of cases and pMCI in 45%, compared with women who experience sMCI in 50% of cases and pMCI in 50%. Notably, 58% of the ADNI2 cohort is male and 42% female. In comparison with 70% and 30% of female subjects, respectively, sMCI occurs in 75% of cases and pMCI in 25% of patients for male subjects. There were 45% female and 55% male in the ADNI-GO cohort. Compared with male patients, who experience sMCI in 89% of cases and pMCI in 11%, female subjects experience sMCI in 95% of cases and pMCI in 5%.

The study assessed the dataset’s performance across various cohorts, accounting for a variable number of slices and including both male and female participants. Each cohort underwent 5 separate runs of the experiments using 5 distinct datasets with slices of 5, 15, 20, 30, and 40 to examine the optimal number of slices that can be used. The performance of the model using a variable number of slices is shown in Table 4.

**Table 4.** The evaluation of the model’s performance across various cohorts and slices using 5 distinct datasets with slices of 5, 15, 20, 30, and 40 to examine the optimal number of slices that can be used, considering both gender groups.

Cohorts	Slices	ACC (%)	F1	SD
ADNI1	5 slices	92.19	0.92	±14.77
	15 slices	91.15	0.91	±15.55
	20 slices	91.16	0.91	±14.93
	30 slices	90.46	0.89	±15.76
	40 slices	89.89	0.89	±15.14
ADNI2	5 slices	92.94	0.88	±12.02
	15 slices	92.57	0.87	±12.52
	20 slices	90.81	0.83	±12.79
	30 slices	91.22	0.83	±11.68
	40 slices	90.20	0.82	±12.60
ADNI-GO	5 slices	96.78	0.98	±7.38
	15 slices	96.87	0.96	±9.39
	20 slices	96.16	0.96	±9.46
	30 slices	97.22	0.97	±8.15
	40 slices	95.79	0.95	±9.87

SD: standard deviation; ADNI: Alzheimer’s Disease Neuroimaging Initiative; ADNI-GO: Alzheimer’s Disease Neuroimaging Initiative-Grand Opportunities; ACC: accuracy.

With five slices, the F1 score for the ADNI1 cohort was 0.92. However, as the number of slices gradually increased, the performance metrics showed a noticeable decline. The F1 score dropped to 0.91 at 15 and 20 slices, respectively. The F1 scores were 0.89 when the slice size was increased further to 30 and 40. While the ADNI2 cohort showed an initial F1 score of 0.88 with 5 slices, however, as the number of slices increased, this performance declined, with the F1 score falling to 0.83 at 40 slices. The ADNI-GO cohort displayed strong results with a commendable F1 score of 0.98 at 5 slices. However, this performance declined as the number of slices increased, with the F1 score dropping to 0.95 at 40 slices.

In the experiments, we examine the effect of gender on the model outcomes using 30 2D slices per subject for each cohort. The experiments were conducted three times for each cohort, producing three distinct gender-specific results for male, female, and combined (male and female) datasets. The outcomes of our experiments, shown in Table 5, analyze the model’s performance across various cohorts and gender categories. We observed differences in the F1 scores between gender groups. The model’s F1 scores for males and females in ADNI1 are 0.89 and 0.93, respectively. The F1 scores of the ADNI2 model are nearly the same for both males and females. Notably, the model showed disparities in the ADNI-GO cohort, achieving F1 scores of 1 and 0.78 for females and males, respectively. The model maintained competitive performance in combined cohorts, with F1 scores of 0.84 for males and 0.85 for females, respectively. Furthermore, when we considered combined genders within each cohort, we found consistent performance, indicating the robustness of our model.

**Table 5.** Assessing the model’s performance across various cohorts and gender groups using 30 2D slices for each subject in each cohort, highlighting how gender affects the model’s ability to predict accurately.

Cohorts		ADNI1			ADNI2			ADNIGO			Combined cohorts		
Subjects	Gender	Male (67%)/female (33%)	Male	Female	Male (58%)/female (42%)	Male	Female	Male (55%)/female (45%)	Male	Female	Male (61%)/female (39%)	Male	Female
	sMCI	53%	55%	50%	73%	75%	70%	92%	89%	95%	69%	69%	69%
	pMCI	47%	45%	50%	27%	25%	30%	8%	11%	5%	31%	31%	31%
ACC (%)		90.46	90.84	92.54	91.22	93.96	93.36	97.22	87.64	100	91.45	89.64	90.3
F1		0.89	0.89	0.93	0.83	0.87	0.88	0.97	0.78	1	0.86	0.84	0.85
SD		±15.76	±15.42	±18.11	±11.60	±10.6	±11.32	±8.15	±19.46	±0	±11.12	±12.72	±11.54

ADNI: Alzheimer’s Disease Neuroimaging Initiative; ADNIGO: Alzheimer’s Disease Neuroimaging Initiative Grand Opportunities; sMCI: stable mild cognitive impairment; pMCI: progressive mild cognitive impairment; SD: standard deviation; ACC: accuracy.

## Discussion

This study aims to predict MCI patients who either remained stable or developed AD using a CDTL approach model trained on sMRI scans of healthy and AD individuals. We develop a model that combines transfer learning and a class decomposition method to detect cognitive decline in AD from structural sMRI scans. We test the model with various datasets, using the same hyperparameters in each experiment, to show the efficacy of the suggested approach.

In order to determine which cohorts or gender groups the model performed best on and where the potential for improvement might exist, we evaluate the model performance across various cohorts, genders, and categories of MCI. Results demonstrated that our model performed well across cohorts and is an accurate predictor of conversion to AD within 36 months. Our model showed a robust performance with different variations of imaging protocols and scanners. Variations in sMCI-to-pMCI ratios between cohorts and gender groups had an impact on overall model performance. When using 30 slices for each subject in each cohort, our model achieved an accuracy of 91.45 and an F1 score of 0.86. The classification performance of predicting sMCI versus pMCI using the pretrained network with the four different datasets is reported in Tables 4 and 5.

Table 4 evaluates model performance across various cohorts and slice configurations while considering male and female patients. The F1 scores in the ADNI1 cohort remained consistently high as the number of slices increased from 5 to 40, ranging from 0.89 to 0.92. Likewise, in ADNI2, the model demonstrates stability across different slice numbers. The ADNI-GO cohort was consistent, with F1 scores ranging from 0.95 to 0.98.

Based on the outcomes in Table 4, we selected 30 slices per subject in each cohort when training and testing the model rather than using five slices, even though five performed slightly better in some cases. Using more slices could be beneficial for the following reasons: (1) increasing the number of slices can give more comprehensive anatomical coverage, giving a model better contextual information about structures and abnormalities. This leads to training models that comprehend finer details to improve model accuracy, (2) the model can have better ability to generalize to previously unseen images with more data, leading to a more robust and reliable model. On the contrary, although increasing the number of slices has its benefits, it can also bring more challenges such as memory and processing requirements, and so on. Therefore, selecting the number of slices that is still representative of the data, and gives good diversity with consideration of existing constraints is an important step. In this article, we chose 30 as the number of slices that reasonably attain the balance among the aforementioned factors.

We conducted gender analysis across all cohorts indicate, as shown in Table 5, that: (**1**) the results show that gender has no considerable impact on the performance of the models. For example, the combined gender group typically revealed accuracies between or close to those of the male and female groups, demonstrating that gender has no noticeable impact on performance. For instance, in the ADNI1 cohort, the females group performed accuracy with 92.54% and the males with 90.84%, while the combined gender reached 90.46%. Also, when the data from all cohorts were combined, the accuracy for both male and female groups was nearly the same, around 90%. (**2**) The model’s performance is influenced by differences in sMCI-to-pMCI ratios across cohorts and gender groups. The ratios of sMCI to pMCI varied across cohorts and gender groups, as shown in Figure 4. ADNI1 and ADNI2 have similar sMCI-to-pMCI ratios across male and female groups, reflecting on the performance of both gender groups. For example, in the ADNI1 and ADNI2 cohorts, the distribution of sMCI and pMCI is roughly (55% for males and 50% for females) and (45% for males and 50% for females), respectively. Females performed slightly better than males (92.54% accuracy) in the ADNI1 cohort; male and female accuracy was the same in the ADNI2 cohort. However, in the ADNI-GO cohort, the proportion of sMCI is higher than that of pMCI, with women showing the most noticeable difference, with sMCI ratios (89% for males and 95% for females) and pMCI ratios (11% for males and 5% for females). Females in the ADNI-GO cohort had the highest accuracy, at 100%. However, the F1 score for that category was 0, indicating potential issues, such as the model only predicting one class. The fact that the standard deviation was 0 also confirms this. (**3**) The model has consistent results among ADNI1, ADNI2, ADNI-GO, and the combined cohorts. The combined gender group’s accuracy was 91.45% in combined cohorts, which is comparable with the accuracy of the same gender category in the ADNI1 and ADNI2 cohorts, which were 90.46% and 91.22%, respectively.

We used class decomposition to make learning the class boundaries within a single class easier by revealing latent knowledge. Each class is broken down into its subclasses by the class decomposition process. However, it can be challenging to use class decomposition when there is a large imbalance in the data distribution within binary tasks. As shown in Figure 4, the proportion of sMCI in the ADNI-GO cohort is higher than that of pMCI, at 92% and 8%, respectively. When training the model using ADNI-GO as a separate dataset in our experiments, we faced an issue because the minority class (pMCI) has significantly fewer samples than sMCI. In this situation, the trained model becomes biased toward the majority class when using a few samples from pMCI. This is due to the model’s learning being influenced by the majority class, which could lead to instances of the minority class being incorrectly classified. The minority class’s breakdown into subclusters with few samples was one of several factors that contributed to the performance decline. To address this issue, the minority class, pMCI, was over-sampled; however, this has an impact on the ADNI-GO cohort’s results in comparison with those of the other cohorts, and it is essential to note that we did not use over-sampling when comparing all cohorts together to form the Combined cohorts. Table 5 shows that the ADNI-GO cohort achieved an F1 score of 0.97, whereas other cohorts achieved F1 scores ranging from 0.83 to 0.89, indicating the effect of the over-sampling on the ADNI-GO results. We can see that ADNI-GO achieved 97.22% accuracy in Table 5 but dropped to 89.27% in Table 6. The difference in results occurred when we over-sampled the dataset by gender (Table 5); we separated the male and female datasets and over-sampled each of them based on the ratio of sMCI and pMCI and then combined over-sampled datasets to form the male and female combined dataset, whereas accuracy in Table 6 was caused by over-sampling the ADNI-GO cohort without taking gender into account. To conclude, class decomposition divides complex classes, particularly minority classes, into smaller subclasses to improve classifier performance. However, when datasets have significant class imbalances, the effectiveness of class decomposition is significantly affected by the majority class. Because of this influence, newly created subclasses may be shaped more by the characteristics of the majority class rather than capturing the true nuances of the minority class. Furthermore, over-sampling, which involves artificially increasing the size of the minority class, may change the actual data distribution even more, adding more complexity to the decomposition process.

**Table 6.** Comparison of experimental results with four ADNI datasets.

Cohort	Accuracy (%)	Sensitivity (%)	Specificity (%)
ADNI1	90.46	90.67	90.23
ADNI2	91.22	91.83	89.37
ADNIGO	89.27	84.02	99.04
Combined cohort	91.45	91.02	92.52

ADNI: Alzheimer’s Disease Neuroimaging Initiative; ADNIGO: Alzheimer’s Disease Neuroimaging Initiative Grand Opportunities.

Table 7 shows some notable patterns when evaluating model performance across different cohorts and gender categories. In the ADNI1 cohort, males had a marginally higher AUC point estimate (0.97) than females and the combined gender category, scoring 0.96. The correspondence of the 95% confidence intervals, particularly in males between 0.97 and 0.98, highlights the consistency of this model’s predictions. The ADNI2 cohort had slightly different outcomes. Females outperformed males with an AUC point estimate of 0.98, equaled by the combined gender category. The narrow confidence intervals highlight the consistency of these estimates, with intervals between 0.97 and 0.98 for the combined gender and female categories. The aggregated cohort revealed closely aligned performance between males and females. AUC point estimates of 0.94 and 0.95 and very narrow confidence intervals suggest consistent and similar performance across genders in the combined cohort dataset. These findings highlight the models’ strong predictive power across cohorts and subtle differences in model performance between genders.

**Table 7.** Evaluation of the model’s performance using AUC and 95% confidence interval (CI) across different cohorts and gender groups.

Cohorts	Gender	AUC	95% CI
ADNI1	Male and female	0.96	(0.96, 0.97)
	Male	0.97	(0.97, 0.98)
	Female	0.96	(0.96, 0.97)
ADNI2	Male and female	0.98	(0.97, 0.98)
	Male	0.96	(0.95, 0.96)
	Female	0.98	(0.97, 0.98)
ADNI-GO	Male and female	1.00	(1.00, 1.00)
	Male	0.92	(0.92, 0.93)
	Female	1.00	(1.00, 1.00)
Combined cohorts	Male and female	0.95	(0.95, 0.95)
	Male	0.94	(0.94, 0.95)
	Female	0.95	(0.95, 0.95)

AUC: area under the curve; ADNI: Alzheimer’s Disease Neuroimaging Initiative; ADNI-GO: Alzheimer’s Disease Neuroimaging Initiative-Grand Opportunities.

**Figure 5.**
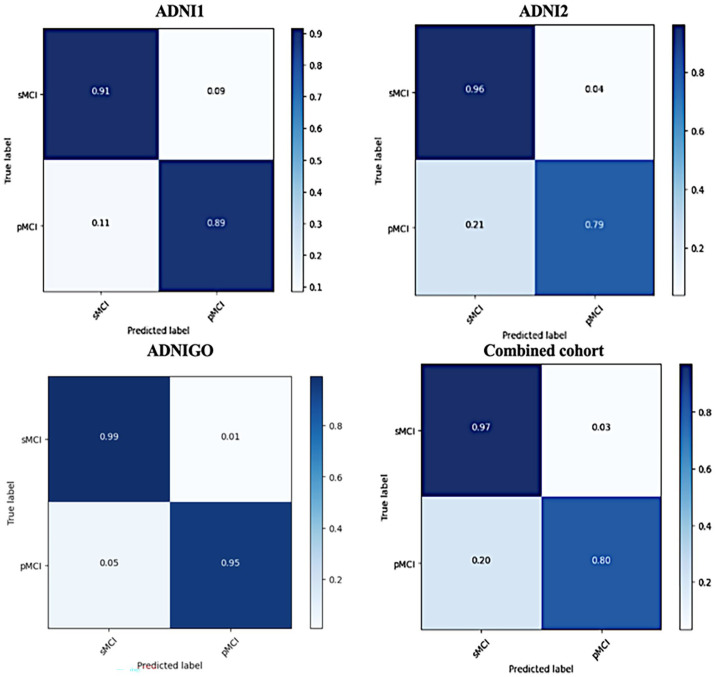
The confusion matrices of using the model with four different ADNI datasets (ADNI-1, ADNI-2, ADNI-GO, and combined cohort).

The outcome of the study indicates the significance of using the CDTL model, which assists in transferring previously learned knowledge from predicting CN versus AD to be used in the MCI-to-AD conversion task. To assess the robustness of the proposed model in detecting the cognitive decline of AD, we evaluate the confusion matrices from the prediction results using different datasets. As shown in Figure 5, the confusion matrices show that using the proposed approach with different datasets predicts the sMCI class significantly better than the pMCI class.

### Comparisons with previous studies

Due to differences in datasets, data preparation strategies, dimensional reduction methods, and measurements between studies, comparing previous research findings has been challenging. Even though the studies may use different experimental setups, the results of the methods are comparable. We compared our model with approaches in Table 8, and the results for these models are what is reported in their articles. We selected these studies because they have been used in the literature for similar or related tasks. In comparison with previous studies (see Table 8), although some studies used 3D images, which provide full-brain volume and analysis, our model outperforms the other approaches in distinguishing pMCI from sMCI within 36 months with an accuracy of 91.45%. Our approach has the following strengths over previous studies: Using transfer learning and class decomposition in the base classifier addresses the issue of limited availability of annotated data, while class decomposition improves model performance by making learning the class boundaries of a dataset simple. Transfer learning was used to transfer knowledge from AD versus CN prediction. Because the differences between sMCI and pMCI are expected to be fewer than those between AD and CN, models trained with AD and HC individuals can be especially effective in distinguishing sMCI and pMCI patients. As a result, the pretrained model performs well in predicting sMCI versus pMCI.

**Table 8.** Comparison of classification performance with state-of-art studies.

Study	Sample size	Input	Data augmentation	Transfer learning	Accuracy (%) (sMCI versus pMCI)
Basaia *et al.*^14^	352 CN 294 AD 763 MCI	3D sMRI	Yes	Yes	75.1
Pan *et al.*^15^	262 CN 237 AD 288 MCI	2D sMRI	Yes	No	62.0
Bae *et al.*^16^	2084 CN 1406 AD 450 MCI	3D sMRI	No	Yes	82.4
Proposed approach	44 CN 45 AD 467 MCI	2D sMRI	No	Yes	91.45

MCI: mild cognitive impairment; sMCI: stable MCI; pMCI: progressive MCI; CN: cognitively normal; AD: Alzheimer’s disease; sMRI: structural magnetic resonance imaging.

## Conclusions and future work

In this study, we examined the robustness of the CDTL model in classifying sMCI and pMCI using data from various ADNI cohorts. Furthermore, we used the model with 2D structural sMRI images to determine whether comparable classification accuracy for two or more cohorts can be obtained and to evaluate the combined cohorts’ prediction accuracy. We employed the CDTL model to predict AD versus CN before it was utilized to predict MCI-to-AD conversion. In addition, we used an entropy-based approach to select the 2D slices with the most informative data.

A limitation in our study pertains to the specific selection criteria we employed. We mainly focused on participants who had follow-up exams 3 years after their baseline assessment. In this regard, our analysis did not consider any baseline-diagnosed MCI subjects who later reverted to a CN status over the 36 months. In addition, we only included people who had MCI at their initial diagnosis and did not change their condition over 3 years, classifying them as having sMCI. On the contrary, individuals diagnosed with pMCI were subjects who changed from MCI to AD during this time. If this classifier were implemented in clinical practice, there would be no way to exclude current probable MCI patients based on their having.

In the future, we aim to combine the model with other data modalities for diagnosing AD, such as PET scans and clinical data.

## References

[1] Alzheimer’s Association. 2018 Alzheimer’s disease facts and figures. Alzheimers Dement 2018;14:367–429

[2] Petersen RC Doody R Kurz A Mohs RC Morris JC Rabins PV Ritchie K Rossor M Thal L Winblad B. Current concepts in mild cognitive impairment. Arch Neurol 2001;58:1985–92 10.1001/archneur.58.12.1985 11735772

[3] Ebrahimighahnavieh MA Luo S Chiong R. Deep learning to detect Alzheimer’s disease from neuroimaging: a systematic literature review. Comput Methods Programs Biomed 2020;187:105242 31837630 10.1016/j.cmpb.2019.105242

[4] Agarwal D Marques G De La Torre-Díez I Franco Martin MA García Zapiraín B Martín Rodríguez F. Transfer learning for Alzheimer’s disease through neuroimaging biomarkers: a systematic review. Sensors 2021;21:7259 34770565 10.3390/s21217259 PMC8587338

[5] Choi H Jin KH. Predicting cognitive decline with deep learning of brain metabolism and amyloid imaging. Behav Brain Res 2018;344:103–9 10.1016/j.bbr.2018.02.017 29454006

[6] Westman E Simmons A Muehlboeck JS Mecocci P Vellas B Tsolaki M Kloszewska I Soininen H Weiner MW Lovestone S Spenger C Wahlund LO. AddNeuroMed and ADNI: similar patterns of Alzheimer’s atrophy and automated MRI classification accuracy in Europe and North America. Neuroimage 2011;58:818–28 10.1016/j.neuroimage.2011.06.065 21763442

[7] Hon M Khan NM. Towards Alzheimer’s disease classification through transfer learning. In: Proceedings of the 2017 IEEE international conference on bioinformatics and biomedicine (BIBM), Kansas, MO, 13–16 November 2017, pp. 1166–1169. New York: IEEE

[8] Kang W Lin L Zhang B Shen X Wu S. Multi-model and multi-slice ensemble learning architecture based on 2D convolutional neural networks for Alzheimer’s disease diagnosis. Comput Biol Med 2021;136:104678 34329864 10.1016/j.compbiomed.2021.104678

[9] Khan NM Abraham N Hon M. Transfer learning with intelligent training data selection for prediction of Alzheimer’s disease. IEEE Access 2019;7:72726–35

[10] Payan A Montana G. Predicting Alzheimer’s disease: a neuroimaging study with 3D convolutional neural networks. 2015, http://arxiv.org/abs/1502.02506

[11] Naz S Ashraf A Zaib A. Transfer learning using freeze features for Alzheimer neurological disorder detection using ADNI dataset. Multimed Syst 2022;28:85–94

[12] Abrol A Bhattarai M Fedorov A Du Y Plis S Calhoun V. Deep residual learning for neuroimaging: an application to predict progression to Alzheimer’s disease. J Neurosci Methods 2020;339:108701 32275915 10.1016/j.jneumeth.2020.108701 PMC7297044

[13] Oh K Chung YC Kim KW Kim WS Oh IS. Classification and visualization of Alzheimer’s disease using volumetric convolutional neural network and transfer learning. Sci Rep 2019;9:18150 31796817 10.1038/s41598-019-54548-6 PMC6890708

[14] Basaia S Agosta F Wagner L Canu E Magnani G Santangelo R Filippi M. Automated classification of Alzheimer’s disease and mild cognitive impairment using a single MRI and deep neural networks. Neuroimage Clin 2019;21:101645 30584016 10.1016/j.nicl.2018.101645 PMC6413333

[15] Pan D Zeng A Jia L Huang Y Frizzell T Song X. Early detection of Alzheimer’s disease using magnetic resonance imaging: a novel approach combining convolutional neural networks and ensemble learning. Front Neurosci 2020;14:259 32477040 10.3389/fnins.2020.00259 PMC7238823

[16] Bae J Stocks J Heywood A Jung Y Jenkins L Hill V Katsaggelos A Popuri K Rosen H Beg MF Wang L. Transfer learning for predicting conversion from mild cognitive impairment to dementia of Alzheimer’s type based on a three-dimensional convolutional neural network. Neurobiol Aging 2021;99:53–64 33422894 10.1016/j.neurobiolaging.2020.12.005 PMC7902477

[17] Ardalan Z Subbian V. Transfer learning approaches for neuroimaging analysis: a scoping review. Front Artif Intell 2022;5:780405 35265830 10.3389/frai.2022.780405 PMC8899512

[18] Tan C Sun F Kong T Zhang W Yang C Liu C . A survey on deep transfer learning. In: 27th international conference on artificial neural networks, 2018, http://arxiv.org/abs/1808.01974

[19] Weiner MW Veitch DP Aisen PS Beckett LA Cairns NJ Cedarbaum J Donohue MC Green RC Harvey D Jack CR Jagust W Morris JC Petersen RC Saykin AJ Shaw L Thompson PM Toga AW Trojanowski JQ. Impact of the Alzheimer’s disease neuroimaging initiative, 2004 to 2014. Alzheimers Dement 2015;11:865–84 10.1016/j.jalz.2015.04.005 PMC4659407 26194320

[20] Haralick RM Dinstein I Shanmugam K. Textural features for image classification. IEEE Trans Syst Man Cybern 1973; SMC-3:610–21

[21] Abbas A Abdelsamea MM Gaber MM. DeTrac: transfer learning of class decomposed medical images in convolutional neural networks. IEEE Access 2020;8:74901–13

[22] Vilalta R Achari MK Eick CF . Class decomposition via clustering: a new framework for low-variance classifiers. In: Proceedings—IEEE international conference on data mining, ICDM, 2003, pp. 673–676, https://ieeexplore.ieee.org/abstract/document/1251005

